# Effect of Serum Ferritin on the Prognosis of Patients with Sepsis: Data from the MIMIC-IV Database

**DOI:** 10.1155/2022/2104755

**Published:** 2022-12-06

**Authors:** Yi-Peng Fang, Hui-Juan Zhang, Zhe Guo, Chun-Hong Ren, Yun-Fei Zhang, Qian Liu, Zhong Wang, Xin Zhang

**Affiliations:** ^1^Laboratory of Molecular Cardiology, The First Affiliated Hospital of Shantou University Medical College, Shantou, Guangdong, China; ^2^Laboratory of Medical Molecular Imaging, The First Affiliated Hospital of Shantou University Medical College, Shantou, Guangdong, China; ^3^Shantou University Medical College, Shantou, Guangdong, China; ^4^Department of Pulmonary and Critical Care Medicine, Beijing Tsinghua Changgung Hospital, Beijing, China; ^5^Department of Liver Intensive Care Unit, Beijing Tsinghua Changgung Hospital, Beijing, China; ^6^International Medical Service Center, The First Affiliated Hospital of Shantou University Medical College, Shantou, Guangdong, China; ^7^Tianjin Hospital of Tianjin University, Tianjin, China; ^8^Department of Cardiology, The Affiliated Hospital of Binzhou Medical University, Binzhou, Shandong, China; ^9^Department of General Practice Medicine, Beijing Tsinghua Changgung Hospital, Beijing, China

## Abstract

**Background:**

The present study aimed to investigate the prognostic value of serum ferritin in critically ill patients with sepsis by using the MIMIC-IV database.

**Methods:**

Data were extracted from the MIMIC-IV database. Adult patients who met the sepsis-3 criteria and had the test of ferritin were included. Patients were divided into subgroups according to the initial serum ferritin. The association between initial serum ferritin and in-hospital mortality was performed by using Lowessregression, logistic regression, and ROC analysis. Subgroup analysis was used to search for the interacting factors and verify the robustness of the results.

**Results:**

Analysis of the 2,451 patients revealed a positive linear relationship between serum ferritin and in-hospital mortality. Patients with high-ferritin had a higher risk of in-hospital mortality, but no significant association was found in the low-ferritin subgroup compared with those whose ferritin was in the normal reference range. Serum ferritin had moderate predictive power for in-hospital mortality (AUC = 0.651), with an optimal cut-off value of 591.5 ng/ml. Ferritin ≥591.5 ng/ml acted as an independent prognostic predictor of in-hospital mortality, which increased the risk of in-hospital mortality by 119%. Our findings were still robust in subgroup analysis, and acute kidney injury and anemia were considered interactive factors.

**Conclusion:**

High-level serum ferritin was an independent prognostic marker for the prediction of mortality in patients with sepsis. Further high-quality research is needed to confirm the relationship between ferritin and the prognosis of septic patients.

## 1. Introduction

Sepsis is life-threatening organ dysfunction caused by infection, which is a fatal disease with a prolonged hospital stay, high morbidity, and mortality rate [1]. Therefore, sepsis is a significant public health problem as more than 19 million patients are diagnosed with sepsis each year and about 40% of septic patients are rehospitalized within their first 90 days after discharge [2]. Although international Surviving Sepsis Campaign guidelines have been regularly updated to direct standardized therapy of sepsis, the treatment of sepsis remains challenging for clinicians, and the mortality of sepsis remains high [3]. The complex pathophysiological processes and untimely intervention are significant contributors to the poor outcome of sepsis. Timely diagnosis and evaluation of the condition of sepsis are key to early treatment and intervention, which are considered crucial aspects for improving the prognosis of septic patients [3]. Some scoring systems, such as sequential organ failure assessment (SOFA), acute physiology and chronic health evaluation II (APACHEII), are widely used in the diagnosis of sepsis and predicting the risk of death for sepsis in clinical practice. All of them are effective but remain too complex and time-consuming due to the inclusion of too many parameters. It is of great significance to find some effective and convenient-to-use biomarkers for the diagnosis and prognosis of sepsis.

Excessive inflammatory response secondary to the dysregulated host response to infection is the core pathogenesis of sepsis in the development of organ damage [4]. Iron is essential for almost all organisms and is required by cells for metabolic needs and specialized functions. Except for acting as the central medium of hemoglobin and myoglobin for oxygen binding, iron also plays key roles in many metabolic processes of both the host and pathogen [5]. Maintaining iron homeostasis of the human body is essential for basic metabolism. Serum ferritin is generally considered a good indicator of iron stores under most circumstances. Meanwhile, serum ferritin is also known as an acute phase reactant, which is regulated either transcriptionally or posttranscriptionally by proinflammatory cytokines. Several recent studies have shown that iron metabolism parameters could be used as prognostic markers in critical patients and septic patients [6–8]. However, due to the heterogeneity of the disease and the participants, further investigations are still needed to explore the feasibility of using iron metabolism parameters as biomarkers to predict the outcomes of septic patients. Although most published studies establish a relationship between the elevation of ferritin and the poor outcome, its clinical applications are still limited and need further evaluation, especially a search for the optimal cut-off value.

In the present study, we tried to investigate the association between serum ferritin and clinical outcomes in septic patients by using the Medical Information Mart for Intensive Care IV (MIMIC-IV) database including over 76,000 critical care patients' admission of 53,569 patients.

## 2. Materials and Methods

### 2.1. Study Design

This was a single-center retrospective cohort study. All data were obtained from the MIMIC-IV database (version 2.0). The MIMIC-IV database is the single center, global, publicly available repository for structural data of critically ill patients from 2008 to 2019 in Beth Israel Deaconess Medical Center (Boston, Massachusetts). Version 2.0 is the latest version of MIMIC-IV, which could be obtained on the PhysioNet freely (Johnson et al., MIMIC-IV (version 2.0). PhysioNet. https://doi.org/10.13026/7vcr-e114) [9]. This database was approved by the Institutional Review Boards (IRBs) of the Massachusetts Institute of Technology (MIT). The author FYP, who passed the examination of the National Institutes of Health (NIH) web-based course named “Protecting Human Research Participants” and obtained the certification (certification number 43025968), was responsible for all the data extraction. Data were extracted by structured query language with pgAdmin4 and PostgreSQL 9.6.

### 2.2. Selection of Septic Patients

**Figure 1:**
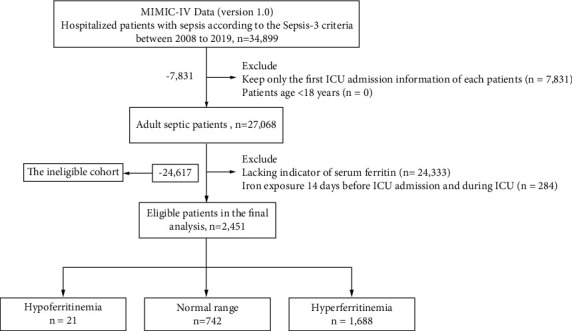
The flow chart.

In the present study, Third International Consensus Definitions for Sepsis and Septic Shock (Sepsis-3) criteria were adapted as the benchmark of sepsis diagnosis [1]. The ID list of septic patients was obtained from the structured view. In short, infectious patients with SOFA score ≥2 were identified as septic patients. There were a total of 34,899 patients diagnosed with sepsis admitted to the ICU department. For patients who were readmitted to the hospital, only the first hospitalization and ICU-admitted information were kept. Patients less than 18 years and those with lack of serum ferritin parameters were excluded. In order to minimize the effects of iron supplements, patients with iron supplement exposure 14 days before and during the ICU admission were excluded. Ultimately, 2,451 eligible patients were included in the final analysis (Figure 1).

### 2.3. Variable Extraction

Demographic information and type of admission were obtained from the admission table, patient table, and ICU detail structured view. Comorbidities were identified on the basis of the recorded ICD-9 codes shown in Table S1. The SOFA score was used to evaluate the severity of sepsis. The value of laboratory findings and vital signs first charted after sepsis diagnosis were collected. Serum ferritin was obtained from the lab-events' table, using the item-id such as “50924.” The normal range of serum ferritin is 30–400 ng/mL for males and 13–150 for females in the MIMIC-IV database. Thus, patients were divided into three groups according to serum ferritin, and the outcomes were further compared among those three groups: the low-ferritin group, normal reference range (NRR) group, and high-ferritin group.

The primary outcome of this study was in-hospital mortality. Secondary outcomes included 28-day mortality, 90-day mortality, ICU mortality, length–of-hospital stay (hospital-LOS), length-of-ICU stay (ICU-LOS), acute kidney injury (AKI), vasopressor using, and the SOFA score. 28-day and 90-day mortality were defined as the number of deaths in the first 28 days and 90 days after initial ICU admission. Hospital-LOS and ICU-LOS were defined as the days spent in the hospital and ICU department. The definition of AKI was based on the Kidney Disease Improving Global Outcomes (KDIGOs) guideline [10]. Vasopressor using was defined when patients had medication records, including norepinephrine, epinephrine, dopamine, and dobutamine, within 24 hours of sepsis diagnosis.

### 2.4. Management of Abnormal Values and Missing Data

The outliers were adjusted by the winsor2 command with the threshold range from 1 to 99. The indicators with more than 25% missing values were removed from the final analysis. Mean or median was used to replace the missing values in indicators with missing data less than 10%. The linear regression method was used to predict and replace the missing values in the remaining data (10% to 25% missing). The detail of the missing value is shown in Table S2.

### 2.5. Statistical Analysis

Categorical data were presented as numbers and proportions and tested by using chi-square or Fisher's exact test. Numerical data were shown as mean ± standard deviation (SD) or median with an interquartile range (IQR) according to whether the variables are normally distributed. Numerical data were tested by using Student's *t*-test or the Mann–Whitney *U* test. For comparisons among the three groups, one-way ANOVA or the Kruskal–Wallis test was performed. Locally weighted scatterplot smoothing (LOWESS) regression and logistic regression were performed to explore the relationship between serum ferritin and hospital mortality of septic patients. The prognostic significance of serum ferritin and the odds risk (OR) with the 95% confidence intervals (CI) were calculated by using a univariate and multivariate logistics model. The variables with *p* value less than 0.10 in the univariate logistic analysis would be further used in the multivariate logistics regression. Receiver operating characteristic curve (ROC) analysis was performed to evaluate the prognostic value of serum ferritin, using the area under curve (AUC) as the evaluation metrics and further confirming the optimal cut-off value. Subgroup analysis was carried out according to age, gender, the SOFA score (median value = 3), vasopressor using, AKI, pathogen culture, and anemia to assess the robustness and explore the sources of heterogeneity.

All statistical analyses in the present study were performed by using Stata statistical software (version 15.0). A two-tailed test with a *p* value <0.05 was considered statistically significant. Our present manuscript was prepared according to the STROBE statement guidelines [11].

## 3. Results

### 3.1. Patient Characteristics and General Clinical Parameters

Overall, 2,451 septic patients were considered for inclusion into the eligible cohort (shown in Figure 1). Compared with the ineligible cohort, patients included in the final analysis had higher mortality, longer LOS, a higher percentage of AKI development, and vasopressor exposure (Table S3). Some differences were found in baseline characteristics between eligible and ineligible cohorts. The baseline characteristics and clinical parameters of the eligible cohort are provided (Table 1). Patients were subdivided into survival and nonsurvival groups according to the in-hospital survival situation. Patients who died were older (65.78 ± 16.43 vs. 62.32 ± 17.47, *p* < 0.001) compared to survivors; no significant difference was found in gender and the type of admission. Except for anemia, no significant difference was found in other comorbidities. Nonsurvival patients were less likely to have anemia (58.02% vs. 67.52%, *p* < 0.001). The SOFA score was higher in the nonsurvivals compared with the survivals (4 [IQR 3 to 6] vs. 3 [IQR 2 to 5], *p* < 0.001). Laboratory findings and vital signs were significantly different between survivals and nonsurvivals. Nonsurvivals had a higher percentage of AKI development (90.30% vs. 74.15%, *p* < 0.001) and vasopressor exposure (55.25% vs. 33.20%, *p* < 0.001). The culture positivity was 37.82% in nonsurvivals, which was higher than survival patients (32.84%, *p*=0.035). All iron metabolism parameters were significantly different (all *p* < 0.001). Serum ferritin was higher in nonsurvival patients compared to survival patients (892 [IQR: 352 to 2881] vs. 430 [IQR: 181 to 1031], *p* < 0.001).

### 3.2. Clinical Outcomes of Septic Patients in Three Ferritin Categories

After dividing the patients into three groups according to the NRR, the relationship between different ferritin categories and the clinical outcomes were further evaluated (shown in Table 2). These results revealed that higher ferritin was significantly associated with higher in-hospital mortality, ICU mortality, 28-day mortality, and 90-day mortality (all *p* < 0.001). The increasing serum ferritin is also related to the longer hospital duration (*p* < 0.001), the longer ICU duration (*p* < 0.001), the higher SOFA score (*p* < 0.001), higher risks of AKI development (*p* < 0.001), and the higher percentage of vasopressor using during the first 24 h of sepsis diagnosis (*p*=0.021). It is important to note that the increase of serum ferritin, even in the normal range, was also associated with a higher risk of AKI (*p*=0.018), the longer duration time of hospital (*p*=0.012), and ICU (*p*=0.008), compared with the low-ferritin group. No significant difference in mortality was found between the low-ferritin group and the NRR group (all *p* > 0.05).

### 3.3. Serum Ferritin and In-Hospital Mortality in Septic Patients

**Figure 2:**
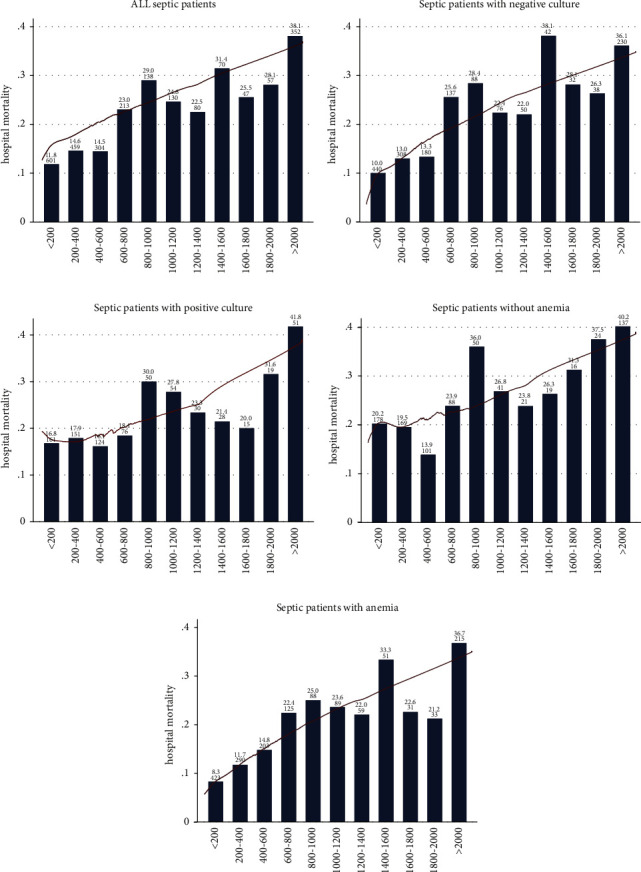
Association between initial serum ferritin and the in-hospital mortality of septic patients. (a) Represents the result obtained from the entire cohort. (b-e) Show the result in those with or without positive culture and anemia. A nearly positive linear relationship was found in those figures, especially in the entire cohort (a), the negative culture cohort (b), and the nonanemic cohort (e). The legends on the top of the bar mean the in-hospital mortality (%) and the number of patients included.

Figure 2 shows the relationship between ferritin and in-hospital mortality for septic patients by using the LOWESS smoothing technique. A nearly linear relationship was found in all septic patients, especially in those with anemia and those without positive culture. It seemed that the higher level of ferritin was associated with the higher mortality of septic patients. However, the relationship was less clear for those with a positive culture and those without anemia (Figures 2(b)–2(e)).

**Figure 3:**
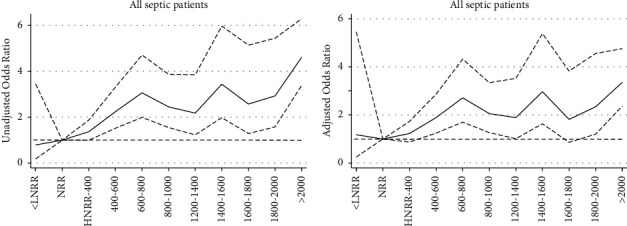
The unadjusted (a) and adjusted odds ratios (ORs) of in-hospital mortality with patients in normal reference range (NRR) as the reference in the entire cohort of septic patients. This figure shows that high ferritin was associated with a higher risk of in-hospital mortality in septic patients. The decrease in serum ferritin had no influence on the risk of in-hospital mortality. NRR for males = 30–400 ng/mL; NRR for females = 13–150 ng/mL; LNRR: the lower limit of NRR; UNRR: the upper limit of NRR.

To further explore the effect of abnormal change of serum ferritin on in-hospital mortality, serum ferritin was categorized into eleven groups. All patients were categorized according to the NRR, and those in the high-ferritin group were further grouped at 200 ng/ml intervals. A logistic regression model was performed to evaluate the relationship between ferritin and the risk of in-hospital mortality with the NRR subgroup as the reference. As shown in Figure 3(a), the elevation of ferritin was associated with increased in-hospital mortality in septic patients with the unadjusted OR ranging from 1.37 (95% CI 1.00 to 1.37, *p*=0.05) to 4.61 (95% CI 3.38 to 6.29, *p* < 0.001). The relationship between ferritin and mortality no longer existed in the low-ferritin subgroup (unadjusted OR 0.79, 95% CI 0.18 to 3.45, and *p*=0.754). In order to remove the effects of confounding factors, a multivariate regression analysis was conducted to identify the independent factors for serum ferritin on in-hospital mortality. The variables with *p* value less than 0.10 in the univariate logistic analysis and gender were included in the present multivariate regression analysis (shown in Table S4). The trend of adjusted OR is shown in Figure 3(b). These trends persisted after adjusting for potential confounders, but the statistically significant difference disappeared in the interval from HNRR to 400 ng/ml (adjusted OR 1.23, 95% CI 0.84 to 1.73, and *p*=0.236) and the interval from 1600 ng/ml to 1800 ng/ml (adjusted OR 1.82, 95% CI 1.82 to 3.82, and *p*=0.116). Low ferritin was not an independent risk factor in the present model (adjusted OR 1.18, 95% CI 0.25 to 5.46, and *p*=0.833).

### 3.4. Predictive Value of Serum Ferritin and High Ferritin for In-Hospital Mortality

**Figure 4:**
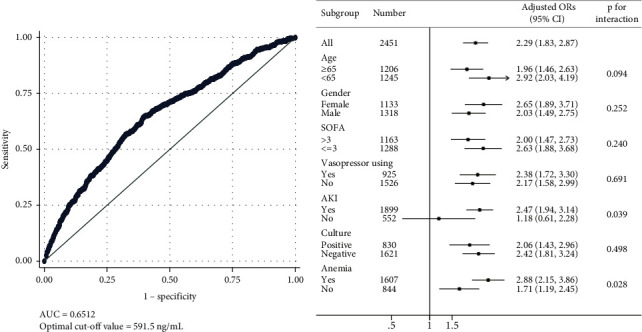
Receiver operating characteristic curves for serum ferritin in the entire cohort of septic patients (a). Initial serum ferritin had a moderate prediction capability with AUC = 0.651 and the optimal cut-off value = 591.5 ng/mL. The adjusted odds ratios (ORs) of ferritin ≥591.5 ng/mL for in-hospital mortality in the entire cohort and different subgroups (b). Ferritin ≥591.5 ng/mL was an independent risk factor of in-hospital mortality. AKI and anemia were the significant interactive factors. SOFA score: the sequential organ failure assessment; AKI: acute kidney injury.

The predictive value and the optimal cut-off value of serum ferritin were calculated for all subgroups in the entire cohort by using ROC analysis (Figure 4(a)). The result showed that serum ferritin had moderate predictive power (AUC = 0.651), and the optimal cut-off value was 591.5 ng/ml. As shown in Table 3, ferritin ≥591.5 ng/ml was an independent predictor of in-hospital mortality of septic patients (adjusted OR 2.29, 95% CI 1.83 to 2.87, and *p* < 0.001). It meant that patients with serum ferritin higher than 591.5 ng/ml were associated with an increase of 119% risk in in-hospital mortality. Elderly, lower temperature, higher respiratory rate, higher WBC, higher lactate, lower hemoglobin, those with SOFA score ≥3, with anemia, vasopressor use, and AKI development were also significantly related to the increased risk of in-hospital mortality (all *p* < 0.05). Subgroup analysis was performed by using the same model to confirm the robustness of our findings and to find potential interactive factors (Figure 4(b)). Our results showed that ferritin ≥591.5 ng/ml performed well in all subgroups except for those without AKI development (adjusted OR 1.18, 95% CI 0.61 to 2.28, and *p*=0.631). AKI and anemia were determined as the significant interactive factors (*p* for interaction = 0.039 and 0.028).

## 4. Discussion

In this retrospective cohort study, we indicated that the elevation of serum ferritin was significantly associated with poor outcomes in septic patients. High ferritin was associated with higher mortality, longer hospital and ICU duration, higher risk of AKI development, and vasopressor using. A positive linear correlation was found between serum ferritin and in-hospital mortality of septic patients. Serum ferritin ≥591.5 ng/ml was an independent predictor of the in-hospital mortality of septic patients, which could increase the in-hospital mortality risk by 119%. Our present study did not reveal that the decrease in ferritin is a risk factor for mortality. AKI and anemia were identified as the significant interactive factors. Our data provided more positive evidence about the effect of serum ferritin on the risk of mortality and prognosis in septic patients, which may further facilitate the clinical application of serum ferritin as a biomarker in diagnosis and prognosis of sepsis.

The balance of iron metabolism is essential to maintaining various metabolisms of humans. Serum ferritin concentration is a useful biomarker reflecting the status of iron stores [12]. A low level of ferritin indicates an iron deficiency, while an elevated ferritin level points to iron overload in the absence of inflammation [12, 13]. Meanwhile, ferritin has been considered an acute phase reactant and could increase significantly in both infectious and noninfectious inflammatory reactions [14]. Recently, the relationships between diseases and ferritin are gaining growing attention. The abnormal changes of ferritin are regarded as diagnostic and prognostic biomarkers of cancer, connective tissue disorders, systemic inflammatory disease, and even the global epidemic of COVID-19 [15–18].

Sepsis has an extremely high morbidity and fatality rate, and seeking effective diagnostic and prognostic indicators of sepsis has always been a topic of considerable interest. The prognostic value of serum ferritin on all-cause mortality in critical patients and septic patients was reported previously [6–8]. Patients with sepsis have higher ferritin levels than those with other diagnoses in ICU departments, while the ferritin level is even higher in the septic shock subgroup [6]. A positive correlation is observed between ferritin and the SOFA score [7]. For the elderly cohort with hyperferritinemia, patients with sepsis or solid malignancy have a worse prognosis than those with other diagnoses [19]. Consistently, we found that there was an obvious iron metabolism imbalance in the present sepsis cohort. Nonsurvival patients had a higher concentration of serum iron, ferritin, and transferrin saturation but a lower level of transferrin, which were consistent with the previous findings [7]. There was a nearly linear relationship between serum ferritin and in-hospital mortality. We further revealed that 591.5 ng/mL had the strongest ability to identify survival and nonsurvival patients during hospitalization. Ferritin concentration exceeding 591.5 ng/mL acted as an independent prognostic predictor of sepsis, and our key findings were still robust in the subgroup analysis. The decrease in serum ferritin had no influence on the mortality of septic patients in the present study, which was not consistent with a previous report about children with severe sepsis and septic shock [20]. In this previous study, children with ferritin less than 200 ng/ml had a higher mortality rate compared with those whose ferritin ranged from 200 to 500 ng/ml (23% vs. 9%). This difference between our study and the previous one could be explained in part by the small sample, different cut-off values, and participants. The real relationship between low-ferritin and NRR should be further confirmed by future studies with a larger sample size. What is more, it is also important to be aware that there were significant differences in baseline information and clinical outcomes between eligible and ineligible subgroups, indicating that those patients included in the final analysis might not be fully representative of the entire sepsis cohort of the MIMIC-IV database. We admitted that potential selection bias and small study bias might have an unknown impact on the present results.

Interestingly, the predictive value was lost in the subgroups without AKI development, but it showed the same tendency with the overall cohort. The interactive effects of AKI and anemia were statistically significant in the present study. AKI is one of the common complications of sepsis. Roughly, one-third of septic patients may develop AKI [21]. Meanwhile, AKI is considered a risk factor for sepsis development. The incidence of sepsis is about 40% in critically ill patients with AKI [22]. The serum ferritin level is considered an effective biomarker in predicting the development of AKI and the recovery of renal function [23, 24]. Although the main function of ferritin is to regulate iron metabolism, some preclinical studies suggested that the effect of ferritin on the kidney is independent of iron loading and may not be limited to iron sequestration [25, 26]. The heavy (FtH) and light (FtL) chains of ferritin are identified as the key regulators of kidney tissue. As Zarjou et al. reported, the loss of FtH from the myeloid compartment contributes to the abrogation of cytokine storm and also significantly protects against septic AKI and improves outcomes [27]. FtH expressed in renal proximal tubules is critical in mediating the tolerance against infection and AKI [28]. The overexpression of FtL can inhibit the inflammatory reaction, reduce organ injury, and promote the survival of infectious mice by inhibiting the activation of the NF-*κ*B pathway [27]. Due to the loss of the predictive value in the nonAKI subgroup, we have concerns with the false positive outcome lead by AKI. Anemia is the key confounder of the present study, especially iron deficiency anemia. Since the decrease of ferritin is the crucial diagnostic criterion of iron deficiency anemia, it is obviously inappropriate to make the diagnosis of iron deficiency anemia in patients with increased ferritin, even using the ICD code in the present study. Anemia was used as a surrogate in the present study. Fortunately, our findings were robust in both anemic and nonanemic subgroups. As a nonspecific biomarker, many influencing factors, including growth hormone, hypoxia, anemia, and endoplasmic reticulum stress, have a great influence on the level of ferritin. Interpretation of the result should be cautious, especially in those combined with diseases that may affect iron metabolism.

Serum ferritin is often increased during sepsis; however, the contributory role of ferritin in sepsis development and progression still could not be established in clinical studies. The following mechanisms may explain the biological relationship between ferritin and sepsis. The first thing to note is the influence of inflammatory factors and acute-phase proteins on erythrocyte damage. Erythropoiesis is known as the key reason for mediating the elevation of circular iron levels, and bacterial proliferation has been shown to be driven by iron sufficiency and suppressed by iron starvation in the preclinical model [29, 30]. In the iron-overloaded mice model, septic mice have an increased susceptibility to infection and higher mortality in the septic model [31, 32]. Since the proliferation of bacteria depends on iron, the host tends to reduce the circular iron levels that can be considered a defense mechanism to limit bacterial growth and resist infection [33]. This effect is mediated by hepcidin, which can effectively decrease intestinal iron absorption and promote macrophages to engulf iron. Ferritin is an important storage protein for iron. The marked reductions in circulating iron mediated by hepcidin can effectively increase the expression of ferritin. What is more, the damage to hepatocytes may result in the release of ferritin into circulation, which is the principal site for the storage of ferritin [34, 35]. Hepatocyte injury also leads to the abnormal synthesis and secretion of hepcidin, which also contributes to the abnormal expression of ferritin [34]. Ferritin is regarded as a protective factor. As McCullough K reported, serum ferritin and FtL can prevent hyperinflammation during sepsis, which is associated with the decrease of NF-*κ*B activation [27, 36]. Furthermore, although reducing circulating iron can effectively reduce bacterial proliferation, iron toxicity, and iron-related oxidative stress, the adverse effects of iron accumulation are also significant. Iron accumulation may lead to cell death, which is known as ferroptosis. This is a newly established type of iron-dependent cell death resulting from iron accumulation and lipid peroxidation [37, 38]. The increased iron loading in macrophages may inhibit the ability to phagocytize and kill pathogens [39]. The effect of FtH and light FtL on sepsis was discussed previously. It is worth noting that an autosomal dominant syndrome named FtL hyperferritinemia may confuse our judgment [36]. Patients with FtL hyperferritinemia have an increased level of intracellular FtL and serum ferritin, but their hyperferritinemia is not associated with inflammatory reactions. The process of iron metabolism during inflammation and infection is very complicated; the relationship between ferritin and sepsis needs to be further explored in the future.

This study has a few limitations. Firstly, due to the nature of the retrospective study, the potential confounders might have unpredictably influenced our conclusion. Secondly, the currently used diagnostic criterion for sepsis was sepsis-3 criteria published in 2016, but the present cohort was constructed for patients from 2008 to 2019. Since the rapid development of sepsis guidelines may bring a great impression on the prognosis of patients with sepsis, using a cohort from the database constructed several years ago may cause assessment bias. Thirdly, the sample size of the present study is still not large enough, especially in subgroup analyses. Some septic patients who were excluded for lacking ferritin results or having iron supplements exposure may lead to selection bias. The bias of small sample size and selection bias should not be ignored. More careful attention should be paid to interpreting the result, especially in some subgroups. Fourthly, as a dynamically changed biomarker, the different trajectories of ferritin may have a different impact on the relationship between ferritin and mortality. Trajectory analysis would be a better method to confirm their relationship. Some sophisticated models should be used to further assess the effect of ferritin trajectories on outcomes in septic patients [40]. High-quality prospective cohort studies and trajectory analysis need to be performed to solve the previously mentioned problems.

## 5. Conclusion

In conclusion, our findings in the present study indicated that a higher level of serum ferritin was associated with a higher risk of mortality in critical patients with sepsis. Serum ferritin may be a potentially useful prognostic biomarker for septic patients in the ICU department, but further large-sample prospective studies are needed to confirm the present finding.

## Figures and Tables

**Table 1:** Comparisons of information between survivors and nonsurvivors.

	Survivals (*n* = 1,946)	Nonsurvivals (*n* = 505)	*p* value
Age (years)	62.32 ± 17.47	65.78 ± 16.43	**<0.001**
Male (%)	1,039 (53.39)	279 (55.25)	0.456
Emergency admissions (%)	1,641 (84.33)	438 (86.73)	0.179
CHD (%)	400 (20.55)	107 (21.19)	0.754
Hypertension (%)	659 (33.86)	166 (32.87)	0.674
HF (%)	604 (31.04)	168 (33.27)	0.337
Diabetes (%)	562 (28.88)	144 (28.51)	0.872
CKD (%)	447 (22.97)	125 (24.75)	0.399
Anemia (%)	1,314 (67.52)	293 (58.02)	**<0.001**
HR (bpm)	91.08 ± 20.68	92.91 ± 20.49	0.076
RR (cpm)	20.45 ± 5.98	21.78 ± 6.47	**<0.001**
MBP (mmHg)	75.48 ± 15.47	73.13 ± 17.77	**0.002**
SpO_2_ (%)	98 (95, 100)	97 (95, 100)	**0.026**
Temperature (°C)	36.96 ± 0.83	36.77 ± 0.88	**<0.001**
WBC (^*∗*^10^9^/L)	10.8 (7.2, 15.2)	12.4 (7.9, 17.9)	**<0.001**
Hemoglobin (g/dL)	9.73 ± 1.98	9.60 ± 2.04	0.215
Creatinine (mmol/L)	1.1 (0.7, 2.0)	1.5 (1.0, 2.5)	**<0.001**
Lactates (mmol/L)	1.5 (1.2, 2.0)	2.2 (1.5, 3.0)	**<0.001**
SOFA score	3 (2, 5)	4 (3, 6)	**<0.001**
AKI (%)	1,443 (74.15)	456 (90.30)	**<0.001**
Vasopressor use (%)	646 (33.20)	279 (55.25)	**<0.001**
Culture positive (%)	639 (32.84)	191 (37.82)	**0.035**
Respiratory system (%)	261 (13.41)	92 (18.22)	**0.006**
Urine system (%)	244 (38.18)	57 (29.84)	**0.035**
Blood system (%)	109 (5.60)	59 (11.68)	**<0.001**
Other system (%)	189 (9.71)	44 (8.74)	0.495
Iron metabolism			
Serum iron (*μ*g/dL)	36 (20, 63)	53 (26, 85)	**<0.001**
Transferrin (mg/dL)	153 (122, 187)	128 (98, 162)	**<0.001**
TIBC (*μ*g/dL)	199 (159, 243)	168 (128, 209)	**<0.001**
Ferritin (ng/mL)	430 (181, 1031)	892 (352, 2281)	**<0.001**

CHD: coronary heart disease; HF: heart failure; CKDs: chronic kidney diseases; HR: heart rate; RR: respiratory rate; MBP: mean blood pressure; WBC: white blood cell; SOFA score: the sequential organ failure assessment; ICU: intensive care unit; LOS: length of stay; AKI: acute kidney injury.

**Table 2:** Unadjusted relationships between serum ferritin groups and clinical outcomes.

	Low-ferritin group	NRR group	High-ferritin group	*p*_1_, *p*_2_ value
Hospital mortality (%)	2 (9.52)	87 (11.73)	416 (24.64)	1.000, **<0.001**
ICU mortality (%)	2 (9.52)	60 (8.09)	304 (18.01)	1.000, **<0.001**
28-day mortality (%)	1 (4.76)	114 (15.36)	431 (25.53)	0.543, **<0.001**
90-day mortality (%)	4 (19.05)	167 (22.51)	602 (35.66)	1.000, **<0.001**
Hospital LOS (days)	5.2 (3.7, 7.9)	9.8 (5.8, 17.5)	14.3 (7.5, 24.4)	**0.012**, **<0.001**
ICU-LOS (days)	1.8 (1.1, 2.3)	3.7 (2.0, 8.5)	6.0 (2.8, 13.4)	**0.008**, **<0.001**
AKI (%)	9 (42.86)	526 (70.89)	1,364 (80.81)	**0.018**, **<0.001**
Vasopressor use (%)	3 (14.29)	252 (33.96)	670 (39.69)	0.177, **0.021**
SOFA score	3 (2, 4)	3 (2, 4)	4 (2, 5)	1.000, **<0.001**

*p*1 value represents the *p* value of comparisons between the low-ferritin group and the NRR group, while *p*2 value represents the *p* value of comparisons between the NRR group and the high-ferritin group. NRR: normal reference range; ICU: intensive care unit; LOS: length of stay; AKI: acute kidney injury; SOFA score: the sequential organ failure assessment.

**Table 3:** Results of log-binomial model analysis.

Variables	B	SE	*z*	Adjusted ORs (95% CI)	*p* value
Hyperferritinemia (%)	0.83	0.26	7.24	2.29 (1.83–2.87)	**<0.001**
Female (%)	−0.12	0.10	−1.03	0.89 (0.71–1.11)	0.303
Age (years)	0.02	0.00	5.25	1.02 (1.01–1.03)	**<0.001**
Anemia (%)	−0.59	0.07	−5.02	0.55 (0.44–0.70)	**<0.001**
Temperature (°C)	−0.20	0.06	−2.97	0.82 (0.72–0.93)	**0.003**
HR (bpm)	0.00	0.00	−0.10	1.00 (0.99–1.01)	0.923
RR (cpm)	0.02	0.01	2.57	1.02 (1.01–1.04)	**0.010**
SpO2 (%)	−0.03	0.02	−1.52	0.97 (0.94–1.01)	0.128
MBP (^*∗*^10^9^/L)	0.00	0.00	−0.83	1.00 (0.99–1.00)	0.408
WBC (^*∗*^10^9^/L)	0.02	0.01	3.06	1.02 (1.01–1.03)	**0.002**
Hemoglobin (g/dL)	−0.12	0.03	−4.20	0.88 (0.83–0.94)	**<0.001**
Lactate (mmol/L)	0.49	0.11	7.60	1.64 (1.44–1.86)	**<0.001**
SOFA score	0.07	0.02	3.08	1.07 (1.02–1.11)	**0.002**
Vasopressor using (%)	0.45	0.19	3.77	1.57 (1.24–1.98)	**<0.001**
AKI (%)	0.73	0.36	4.26	2.08 (1.48–2.90)	**<0.001**
Culture positive (%)	−0.02	0.11	−0.20	0.98 (0.78–1.23)	0.841

CHD: coronary heart disease; HF: heart failure; CKDs: chronic kidney diseases; HR: heart rate; RR: respiratory rate; MBP: mean blood pressure; WBC: white blood cell; SOFA score: the sequential organ failure assessment; AKI: acute kidney injury.

## Data Availability

All datasets used during the present study are publicly available in the MIMIC-IV v2.0 database (https://mimic.physionet.org).

## Supplementary Materials

Supplementary Materials The structured data used in the present study are presented in the Supplemental files S2. The completed STROBE guideline is shown in Supplemental files S3.
