# Supplementary material for: Effect of Serum Ferritin on the Prognosis of Patients with Sepsis: Data from the MIMIC-IV Database
Source: Emerg Med Int. 2022 Dec 6;2022:2104755. doi: 10.1155/2022/2104755 (PMC9747303; doi:10.1155/2022/2104755)
Supplement: Supplementary Materials — The structured data used in the present study are presented in the Supplemental files S2. The completed STROBE guideline is shown in Supplemental files S3. [file 2104755.f1.zip › Supplemental file1 Additional data for present study (1).docx]

**Effect of Serum Ferritin on the Prognosis of Patients with Sepsis data from MIMIC-IV database**

**SUPPLEMENTAL MATERIAL**

**Table S1**. The ICD-9 codes used to screen for complications.

- The ICD codes used to screen for complications, including diabetes (DM), anemia, coronary heart disease (CHD), heart failure (HF), hypertension (HT) and chronic kidney disease (CKD), were shown in TableS1.

**Table S2** Amount of missing data for each variable included in the final analysis.

- The details of missing data were shown in Table S2. The missing percentage of most variables was less than 10%, and they were filled in with the median or mean value. The missing data of lactates was replaced using linear regression.

**Table S3**. The baseline information and outcomes between patients with ferritin examination (eligible cohort) and those without (ineligible cohort).

- There were significant difference of baseline information and clinical outcomes between the eligible cohort and the ineligible cohort (Shown in Table S3). Our eligible cohort, which included in the final study, was younger, and had the lower proportion of men (all *p* < 0.001). Eligible cohort had the lower percentage of CHD, HT and DM, but lower percentage of HF, CKD and anemia (all (*p* < 0.05). Several vital signs and laboratory parameters were significantly different. Eligible cohort had the higher mortality rate (all *p* < 0.001) and longer LOS (all *p* < 0.001). AKI was more common, and vasopressor was also more frequently used in the eligible cohort (all *p* < 0.001). All of them suggested that selective bias may exist in present study. Our findings should be interpreted with caution.

**Table S4** Table S4 The unadjusted OR for in-hospital mortality of all variables

- The result of univariate logistic analysis was shown in Table S4. The variables with *p*-value less than 0.10 gender were included in present multivariate regression analysis.

This supplementary material has been provided by the authors in order to give readers additional information about their work.

Table S1 The ICD codes used to screen for complications

| DM | | | | Anemia | CHD | HF | HT | CKD |
| --- | --- | --- | --- | --- | --- | --- | --- | --- |
| 24900 | E0861 | E103312 | E113553 | 2800 | 41400 | 39891 | 4010 | 28521 |
| 24901 | E08610 | E103313 | E113559 | 2801 | 41401 | 40201 | 4011 | 40300 |
| 24910 | E08618 | E103319 | E11359 | 2808 | 41402 | 40211 | 4019 | 40301 |
| 24911 | E0862 | E10339 | E113591 | 2809 | 41403 | 40291 | 40501 | 40310 |
| 24920 | E08620 | E103391 | E113592 | 2810 | 41404 | 40401 | 40509 | 40311 |
| 24921 | E08621 | E103392 | E113593 | 2811 | 41405 | 40403 | 40511 | 40390 |
| 24930 | E08622 | E103393 | E113599 | 2812 | 41406 | 40411 | 40519 | 40391 |
| 24931 | E08628 | E103399 | E1136 | 2813 | 41407 | 40413 | 40591 | 40400 |
| 24940 | E0863 | E1034 | E1137 | 2814 | 4143 | 40491 | 40599 | 40401 |
| 24941 | E08630 | E10341 | E1137X1 | 2818 | 4144 | 40493 | I10 | 40402 |
| 24950 | E08638 | E103411 | E1137X2 | 2819 | I2101 | 4280 | I15 | 40403 |
| 24951 | E0864 | E103412 | E1137X3 | 2822 | I2102 | 4281 | I150 | 40410 |
| 24960 | E08641 | E103413 | E1137X9 | 2823 | I2109 | 42820 | I151 | 40411 |
| 24961 | E08649 | E103419 | E1139 | 2828 | I2111 | 42821 | I152 | 40412 |
| 24970 | E0865 | E10349 | E114 | 2829 | I2119 | 42822 | I158 | 40413 |
| 24971 | E0869 | E103491 | E1140 | 2830 | I2121 | 42823 | I159 | 40490 |
| 24980 | E088 | E103492 | E1141 | 28310 | I240 | 42830 | O16 | 40491 |
| 24981 | E089 | E103493 | E1142 | 28319 | I251 | 42831 | O161 | 40492 |
| 24990 | E09 | E103499 | E1143 | 2839 | I2510 | 42832 | O162 | 40493 |
| 24991 | E090 | E1035 | E1144 | 28409 | I2511 | 42833 | O163 | 5851 |
| 25000 | E0900 | E10351 | E1149 | 28489 | I25110 | 42840 | O169 | 5852 |
| 25001 | E0901 | E103511 | E115 | 2849 | I25111 | 42841 |  | 5853 |
| 25002 | E091 | E103512 | E1151 | 2850 | I25118 | 42842 |  | 5854 |
| 25003 | E0910 | E103513 | E1152 | 2851 | I25119 | 42843 |  | 5855 |
| 25010 | E0911 | E103519 | E1159 | 28521 | I257 | 4289 |  | 5859 |
| 25011 | E092 | E10352 | E116 | 28522 | I2570 | I0981 |  | D631 |
| 25012 | E0921 | E103521 | E1161 | 28529 | I25700 | I110 |  | E0822 |
| 25013 | E0922 | E103522 | E11610 | 2853 | I25701 | I130 |  | E0922 |
| 25020 | E0929 | E103523 | E11618 | 2858 | I25708 | I132 |  | E1022 |
| 25021 | E093 | E103529 | E1162 | 2859 | I25709 | I50 |  | E1122 |
| 25022 | E0931 | E10353 | E11620 | D460 | I2571 | I502 |  | E1322 |
| 25023 | E09311 | E103531 | E11621 | D461 | I25710 | I5020 |  | I12 |
| 25030 | E09319 | E103532 | E11622 | D462 | I25711 | I5021 |  | I120 |
| 25031 | E0932 | E103533 | E11628 | D4620 | I25718 | I5022 |  | I129 |
| 25032 | E09321 | E103539 | E1163 | D4621 | I25719 | I5023 |  | I13 |
| 25033 | E093211 | E10354 | E11630 | D4622 | I2572 | I503 |  | I130 |
| 25040 | E093212 | E103541 | E11638 | D464 | I25720 | I5030 |  | I131 |
| 25041 | E093213 | E103542 | E1164 | D50 | I25721 | I5031 |  | I1310 |
| 25042 | E093219 | E103543 | E11641 | D500 | I25728 | I5032 |  | I1311 |
| 25043 | E09329 | E103549 | E11649 | D508 | I25729 | I5033 |  | I132 |
| 25050 | E093291 | E10355 | E1165 | D509 | I2573 | I504 |  | N18 |
| 25051 | E093292 | E103551 | E1169 | D51 | I25730 | I5040 |  | N181 |
| 25052 | E093293 | E103552 | E118 | D510 | I25731 | I5041 |  | N182 |
| 25053 | E093299 | E103553 | E119 | D511 | I25738 | I5042 |  | N183 |
| 25060 | E0933 | E103559 | E13 | D513 | I25739 | I5043 |  | N184 |
| 25061 | E09331 | E10359 | E130 | D518 | I2575 | I508 |  | N185 |
| 25062 | E093311 | E103591 | E1300 | D519 | I25750 | I5081 |  | N189 |
| 25063 | E093312 | E103592 | E1301 | D52 | I25751 | I50810 |  |  |
| 25070 | E093313 | E103593 | E131 | D520 | I25758 | I50811 |  |  |
| 25071 | E093319 | E103599 | E1310 | D521 | I25759 | I50812 |  |  |
| 25072 | E09339 | E1036 | E1311 | D528 | I2576 | I50813 |  |  |
| 25073 | E093391 | E1037 | E132 | D529 | I25760 | I50814 |  |  |
| 25080 | E093392 | E1037X1 | E1321 | D53 | I25761 | I5082 |  |  |
| 25081 | E093393 | E1037X2 | E1322 | D530 | I25768 | I5083 |  |  |
| 25082 | E093399 | E1037X3 | E1329 | D531 | I25769 | I5084 |  |  |
| 25083 | E0934 | E1037X9 | E133 | D532 | I2579 | I5089 |  |  |
| 25090 | E09341 | E1039 | E1331 | D538 | I25790 | I509 |  |  |
| 25091 | E093411 | E104 | E13311 | D539 | I25791 | I9713 |  |  |
| 25092 | E093412 | E1040 | E13319 | D55 | I25798 | I97130 |  |  |
| 25093 | E093413 | E1041 | E1332 | D550 | I25799 | I97131 |  |  |
| E08 | E093419 | E1042 | E13321 | D551 | I2581 |  |  |  |
| E080 | E09349 | E1043 | E133211 | D552 | I25810 |  |  |  |
| E0800 | E093491 | E1044 | E133212 | D553 | I25811 |  |  |  |
| E0801 | E093492 | E1049 | E133213 | D558 | I25812 |  |  |  |
| E081 | E093493 | E105 | E133219 | D559 | I2582 |  |  |  |
| E0810 | E093499 | E1051 | E13329 | D58 | I2583 |  |  |  |
| E0811 | E0935 | E1052 | E133291 | D588 | I2584 |  |  |  |
| E082 | E09351 | E1059 | E133292 | D589 | T822 |  |  |  |
| E0821 | E093511 | E106 | E133293 | D59 | T8221 |  |  |  |
| E0822 | E093512 | E1061 | E133299 | D590 | T82211 |  |  |  |
| E0829 | E093513 | E10610 | E1333 | D591 | T82211A |  |  |  |
| E083 | E093519 | E10618 | E13331 | D592 | T82211D |  |  |  |
| E0831 | E09352 | E1062 | E133311 | D594 | T82211S |  |  |  |
| E08311 | E093521 | E10620 | E133312 | D598 | T82212 |  |  |  |
| E08319 | E093522 | E10621 | E133313 | D599 | T82212A |  |  |  |
| E0832 | E093523 | E10622 | E133319 | D61 | T82212D |  |  |  |
| E08321 | E093529 | E10628 | E13339 | D610 | T82212S |  |  |  |
| E083211 | E09353 | E1063 | E133391 | D6109 | T82213 |  |  |  |
| E101 | E093531 | E10630 | E133392 | D611 | T82213A |  |  |  |
| E083212 | E093532 | E10638 | E133393 | D612 | T82213D |  |  |  |
| E083213 | E093533 | E1064 | E133399 | D613 | T82213S |  |  |  |
| E083219 | E093539 | E10641 | E1334 | D618 | T82218 |  |  |  |
| E08329 | E09354 | E10649 | E13341 | D6189 | T82218A |  |  |  |
| E083291 | E093541 | E1065 | E133411 | D619 | T82218D |  |  |  |
| E083292 | E093542 | E1069 | E133412 | D62 | T82218S |  |  |  |
| E083293 | E093543 | E108 | E133413 | D63 | T82855 |  |  |  |
| E083299 | E093549 | E109 | E133419 | D630 | T82855A |  |  |  |
| E0833 | E09355 | E11 | E13349 | D631 | T82855D |  |  |  |
| E08331 | E093551 | E110 | E133491 | D638 | T82855S |  |  |  |
| E083311 | E093552 | E1100 | E133492 | D64 | Z951 |  |  |  |
| E083312 | E093553 | E1101 | E133493 | D640 | Z955 |  |  |  |
| E083313 | E093559 | E111 | E133499 | D641 | Z9861 |  |  |  |
| E083319 | E09359 | E1110 | E1335 | D642 |  |  |  |  |
| E08339 | E093591 | E1111 | E13351 | D643 |  |  |  |  |
| E083391 | E093592 | E112 | E133511 | D644 |  |  |  |  |
| E083392 | E093593 | E1121 | E133512 | D648 |  |  |  |  |
| E083393 | E093599 | E1122 | E133513 | D6481 |  |  |  |  |
| E083399 | E0936 | E1129 | E133519 | D6489 |  |  |  |  |
| E0834 | E0937 | E113 | E13352 | D649 |  |  |  |  |
| E08341 | E0937X1 | E1131 | E133521 | D75A |  |  |  |  |
| E083411 | E0937X2 | E11311 | E133522 | P612 |  |  |  |  |
| E083412 | E0937X3 | E11319 | E133523 | P613 |  |  |  |  |
| E083413 | E0937X9 | E1132 | E133529 | P614 |  |  |  |  |
| E083419 | E0939 | E11321 | E13353 |  |  |  |  |  |
| E08349 | E094 | E113211 | E133531 |  |  |  |  |  |
| E083491 | E0940 | E113212 | E133532 |  |  |  |  |  |
| E083492 | E0941 | E113213 | E133533 |  |  |  |  |  |
| E083493 | E0942 | E113219 | E133539 |  |  |  |  |  |
| E083499 | E0943 | E11329 | E13354 |  |  |  |  |  |
| E0835 | E0944 | E113291 | E133541 |  |  |  |  |  |
| E08351 | E0949 | E113292 | E133542 |  |  |  |  |  |
| E083511 | E095 | E113293 | E133543 |  |  |  |  |  |
| E083512 | E0951 | E113299 | E133549 |  |  |  |  |  |
| E083513 | E0952 | E1133 | E13355 |  |  |  |  |  |
| E083519 | E0959 | E11331 | E133551 |  |  |  |  |  |
| E08352 | E096 | E113311 | E133552 |  |  |  |  |  |
| E083521 | E0961 | E113312 | E133553 |  |  |  |  |  |
| E083522 | E09610 | E113313 | E133559 |  |  |  |  |  |
| E083523 | E09618 | E113319 | E13359 |  |  |  |  |  |
| E083529 | E0962 | E11339 | E133591 |  |  |  |  |  |
| E08353 | E09620 | E113391 | E133592 |  |  |  |  |  |
| E083531 | E09621 | E113392 | E133593 |  |  |  |  |  |
| E083532 | E09622 | E113393 | E133599 |  |  |  |  |  |
| E083533 | E09628 | E113399 | E1336 |  |  |  |  |  |
| E083539 | E0963 | E1134 | E1337 |  |  |  |  |  |
| E08354 | E09630 | E11341 | E1337X1 |  |  |  |  |  |
| E083541 | E09638 | E113411 | E1337X2 |  |  |  |  |  |
| E083542 | E0964 | E113412 | E1337X3 |  |  |  |  |  |
| E083543 | E09641 | E113413 | E1337X9 |  |  |  |  |  |
| E083549 | E09649 | E113419 | E1339 |  |  |  |  |  |
| E08355 | E0965 | E11349 | E134 |  |  |  |  |  |
| E083551 | E0969 | E113491 | E1340 |  |  |  |  |  |
| E083552 | E098 | E113492 | E1341 |  |  |  |  |  |
| E083553 | E099 | E113493 | E1342 |  |  |  |  |  |
| E083559 | E10 | E113499 | E1343 |  |  |  |  |  |
| E08359 | E1010 | E1135 | E1344 |  |  |  |  |  |
| E083591 | E1011 | E11351 | E1349 |  |  |  |  |  |
| E083592 | E102 | E113511 | E135 |  |  |  |  |  |
| E083593 | E1021 | E113512 | E1351 |  |  |  |  |  |
| E083599 | E1022 | E113513 | E1352 |  |  |  |  |  |
| E0836 | E1029 | E113519 | E1359 |  |  |  |  |  |
| E0837 | E103 | E11352 | E136 |  |  |  |  |  |
| E0837X1 | E1031 | E113521 | E1361 |  |  |  |  |  |
| E0837X2 | E10311 | E113522 | E13610 |  |  |  |  |  |
| E0837X3 | E10319 | E113523 | E13618 |  |  |  |  |  |
| E0837X9 | E1032 | E113529 | E1362 |  |  |  |  |  |
| E0839 | E10321 | E11353 | E13620 |  |  |  |  |  |
| E084 | E103211 | E113531 | E13621 |  |  |  |  |  |
| E0840 | E103212 | E113532 | E13622 |  |  |  |  |  |
| E0841 | E103213 | E113533 | E13628 |  |  |  |  |  |
| E0842 | E103219 | E113539 | E1363 |  |  |  |  |  |
| E0843 | E10329 | E11354 | E13630 |  |  |  |  |  |
| E0844 | E103291 | E113541 | E13638 |  |  |  |  |  |
| E0849 | E103292 | E113542 | E1364 |  |  |  |  |  |
| E085 | E103293 | E113543 | E13641 |  |  |  |  |  |
| E0851 | E103299 | E113549 | E13649 |  |  |  |  |  |
| E0852 | E1033 | E11355 | E1365 |  |  |  |  |  |
| E0859 | E10331 | E113551 | E1369 |  |  |  |  |  |
| E086 | E103311 | E113552 | E138 |  |  |  |  |  |
|  |  |  | E139 |  |  |  |  |  |

Table S2 Amount of missing data for each variable included in the final analysis

|  | Missing data number | Missing data percentage |
| --- | --- | --- |
| Age | 0 | 0% |
| Male | 0 | 0% |
| Emergency admissions | 0 | 0% |
| CHD | 0 | 0% |
| Hypertension | 0 | 0% |
| HF | 0 | 0% |
| Diabetes | 0 | 0% |
| CKD | 0 | 0% |
| Anemia | 0 | 0% |
| HR | 0 | 0% |
| RR | 0 | 0% |
| MBP | 34 | 1.3% |
| SpO2 | 2 | <0.1% |
| Temperature | 14 | 0.6% |
| WBC | 27 | 1.1% |
| Hemoglobin | 17 | 0.7% |
| Creatinine | 38 | 1.6% |
| Lactates | 511 | 20.8% |
| SOFA score | 0 | 0% |
| Serum iron | 213 | 8.7% |
| Transferrin | 252 | 10.3% |
| TIBC | 253 | 10.3% |
| Ferritin | 0 | 0% |

CHD, coronary heart disease; HF, heart failure; CKD, chronic kidney diseases; HR, heart rate; RR, respiratory rate; MBP, mean blood pressure; WBC, white blood cell; SOFA score, the Sequential Organ Failure Assessment; ICU, intensive care unit; LOS, length of stay; AKI, acute kidney injury

Table S3 The baseline information and outcomes between patients with ferritin examination (eligible cohort) and those without (ineligible cohort)

|  | Eligible cohort  (n =2,451) | Ineligible cohort  (n = 24,617) | *p*-value |
| --- | --- | --- | --- |
| Age (years) | 63.03±17.31 | 67.05±16.14 | **< 0.001** |
| Male (%) | 1,318 (53.77) | 14,267 (57.96) | **< 0.001** |
| Emergency admissions (%) | 2,079 (84.82) | 17,558 (71.32) | **< 0.001** |
| CHD (%) | 507 (20.69) | 7,850 (31.89) | **<0.001** |
| Hypertension (%) | 825 (33.66) | 10,628 (43.17) | **<0.001** |
| HF (%) | 772 (31.50) | 6,951 (28.24) | **0.001** |
| Diabetes (%) | 706 (28.80) | 7,718 (31.35) | **0.009** |
| CKD (%) | 572 (23.34) | 5,273 (21.42) | **0.028** |
| Anemia | 1,607 (65.57) | 10,739 (43.62) | **< 0.001** |
| HR (bpm) | 91.45 ± 20.65 | 87.34 ± 18.94 | **< 0.001** |
| RR (cpm) | 20.73 ± 6.11 | 19.39 ± 5.64 | **< 0.001** |
| MBP (mmHg) | 75.00 ± 15.46 | 74.57 ± 15.05 | 0.184 |
| SpO2 (%) | 98 (95,100) | 98 (96,100) | **< 0.001** |
| Temperature (℃) | 36.92 ± 0.85 | 36.80 ± 0.82 | **< 0.001** |
| WBC (*10^9^/L) | 11.0 (7.3, 15.8) | 11.4 (8.1,15.5) | **0.008** |
| Hemoglobin (g/dL) | 9.70 ± 2.00 | 10.40 ± 1.92 | **< 0.001** |
| Creatinine (mmol/L) | 1.2 (0.8, 2.1) | 1.0 (0.7, 1.5) | **< 0.001** |
| Lactates (mmol/L) | 1.7 (1.2,2.5) | 1.7 (1.2,2.5) | 0.817 |
| SOFA score | 3 (2, 5) | 3 (2, 4) | **< 0.001** |
| Outcomes |  |  |  |
| Hospital mortality (%) | 505 (20.60) | 3,867 (15.71) | **< 0.001** |
| ICU mortality (%) | 366 (14.93) | 2,743 (11.14) | **< 0.001** |
| 28-day mortality (%) | 546 (22.28) | 4,627 (18.80) | **< 0.001** |
| 90-day mortality (%) | 773 (31.54) | 6,142 (24.95) | **< 0.001** |
| Hospital LOS (days) | 12.6 (6.6, 22.7) | 8.2 (5.0, 14.5) | **< 0.001** |
| ICU LOS (days) | 5.0 (2.4, 11.9) | 2.7 (1.4, 5.4) | **< 0.001** |
| AKI (%) | 1,899 (77.48) | 17,463 (70.94) | **< 0.001** |
| Vasopressor use (%) | 925 (37.74) | 6,844 (27.80) | **< 0.001** |

CHD, coronary heart disease; HF, heart failure; CKD, chronic kidney diseases; HR, heart rate; RR, respiratory rate; MBP, mean blood pressure; WBC, white blood cell; SOFA score, the Sequential Organ Failure Assessment; ICU, intensive care unit; LOS, length of stay; AKI, acute kidney injury

Table S4 The unadjusted OR for in-hospital mortality of all variables

| Variables | B | SE | z | Adjusted ORs | *p*-value |
| --- | --- | --- | --- | --- | --- |
| Female (%) | -0.07 | 0.093135 | -0.75 | 0.93(0.76-1.13) | 0.456* |
| Age (years) | 0.01 | 0.003026 | 3.99 | 1.01(1.01-1.02) | **<0.001*** |
| Emergency admissions (%) | 0.19 | 0.17648 | 1.34 | 1.22(0.91-1.62) | 0.18 |
| CHD (%) | -0.04 | 0.101516 | -0.42 | 0.96(0.78-1.18) | 0.674 |
| Hypertension (%) | 0.10 | 0.11785 | 0.96 | 1.11(0.90-1.36) | 0.337 |
| HF (%) | -0.02 | 0.108574 | -0.16 | 0.98(0.79-1.22) | 0.872 |
| Diabetes (%) | 0.04 | 0.127283 | 0.31 | 1.04(0.82-1.32) | 0.754 |
| CKD (%) | 0.10 | 0.12834 | 0.84 | 1.10(0.88-1.39) | 0.399 |
| Anemia (%) | -0.41 | 0.068029 | -3.99 | 0.66(0.54-0.81) | **<0.001*** |
| Temperatur (℃) | 0.15 | 0.021864 | 8.23 | 1.17(1.12-1.21) | **<0.001*** |
| HR (bpm) | -0.27 | 0.046215 | -4.49 | 0.76(0.68-0.86) | **<0.001*** |
| RR (cpm) | 0.00 | 0.002419 | 1.78 | 1.00(1.00-1.01) | **0.076*** |
| SpO2 (%) | 0.03 | 0.008293 | 4.33 | 1.04(1.02-1.05) | **<0.001*** |
| MBP (*10^9^/L) | -0.04 | 0.01505 | -2.84 | 0.96(0.93-0.99) | **0.004*** |
| WBC (*10^9^/L) | -0.01 | 0.003332 | -3.06 | 0.99(0.98-1.00) | **0.002*** |
| Hemoglobin (g/dL) | 0.03 | 0.006298 | 5.42 | 1.03(1.02-1.05) | **<0.001*** |
| Creatinine (mmol/L) | -0.03 | 0.024633 | -1.24 | 0.97(0.92-1.02) | 0.215 |
| Lactate (mmol/L) | 0.06 | 0.03202 | 1.93 | 1.06(1.00-1.12) | **0.054*** |
| SOFA score | 0.68 | 0.111632 | 12.05 | 1.98(1.77-2.21) | **<0.001*** |
| Vasopressor using (%) | 0.91 | 0.252451 | 8.96 | 2.48(2.04-3.03) | **<0.001*** |
| AKI (%) | 1.18 | 0.515796 | 7.4 | 3.24(2.38-4.43) | **<0.001*** |
| Culture positive (%) | 0.22 | 0.129 | 2.11 | 1.24(1.02-1.52) | **0.035*** |

CHD, coronary heart disease; HF, heart failure; CKD, chronic kidney diseases; HR, heart rate; RR, respiratory rate; MBP, mean blood pressure; WBC, white blood cell; SOFA score, the Sequential Organ Failure Assessment; AKI, acute kidney injury; *, variables were considered as the confounding factors and would be adjusted in the logistical analysis
