# Supplementary material for: Effect of Serum Ferritin on the Prognosis of Patients with Sepsis: Data from the MIMIC-IV Database
Source: Emerg Med Int. 2022 Dec 6;2022:2104755. doi: 10.1155/2022/2104755 (PMC9747303; doi:10.1155/2022/2104755)
Supplement: Supplementary Materials — The structured data used in the present study are presented in the Supplemental files S2. The completed STROBE guideline is shown in Supplemental files S3. [file 2104755.f1.zip › Supplemental file3 The completed STROBE guideline of present study (1).pdf]

PDF-XChange Editor
Click to BUY NOW!
www.tracker-software.com

STROBE Statement—checklist of items that should be included in reports of observational studies

Section/item	Item No	Recommendation	Reported on Page Number/Line Number	Reported on Section/Paragraph
**Title and abstract**	1	(a) Indicate the study's design with a commonly used term in the title or the abstract	Page 3/Line 40-44	Abstract/Paragraph 2
		(b) Provide in the abstract an informative and balanced summary of what was done and what was found	Page 3/ Line 40-55	Abstract/Paragraph 2-4
**Introduction**				
Background/ rationale	2	Explain the scientific background and rationale for the investigation being reported	Page 4-5/Line 58-86	Introduction/Paragraph 1-2
Objectives	3	State specific objectives, including any prespecified hypotheses	Page 5/Line 87-89	Introduction/Paragraph 3
**Methods**				
Study design	4	Present key elements of study design early in the paper	Page 5/Line 92-103	Methods/Paragraph 1
Setting	5	Describe the setting, locations, and relevant dates, including periods of recruitment, exposure, follow-up, and data collection	Page 5/Line 92-103	Methods/Paragraph 1
Participants	6	(a) ***Cohort study***—Give the eligibility criteria, and the sources and methods of selection of participants. Describe methods of follow-up<br>***Case-control study***—Give the eligibility criteria, and the sources and methods of case ascertainment and control selection. Give the rationale for the choice of cases and controls<br>***Cross-sectional study***—Give the eligibility criteria, and the sources and methods of selection of participants	Page 6/Line 105-113	Methods/Paragraph 2
		(b) ***Cohort study***—For matched studies, give matching criteria and number of exposed and unexposed<br>***Case-control study***—For matched studies, give matching criteria and the number of controls per case	NA	NA
Variables	7	Clearly define all outcomes, exposures, predictors, potential confounders, and effect modifiers. Give diagnostic criteria, if applicable	Page 6-7Line 115-131	Methods/Paragraph 3-4
Data sources/ measurement	8*	For each variable of interest, give sources of data and details of methods of assessment (measurement). Describe comparability of assessment methods if there is more than one group	Page 6-7Line 115-131	Methods/Paragraph 3-4
Bias	9	Describe any efforts to address potential sources of bias	Page 8/Line 145-148	Methods/Paragraph 6
Study size	10	Explain how the study size was arrived at	Page 6/Line 105-113	Methods/Paragraph 2
Quantitative variables	11	Explain how quantitative variables were handled in the analyses. If applicable, describe which groupings were chosen and why	Page 8/Line 148-152	Methods/Paragraph 6

PDF-XChange Editor Click to BUY NOW! www.tracker-software.com

Statistical methods	12	(a) Describe all statistical methods, including those used to control for confounding	Page 7-8/Line 138-155	Methods/Paragraph 6-7
		(b) Describe any methods used to examine subgroups and interactions	Page 7-8/Line 150-152	Methods/Paragraph 6
		(c) Explain how missing data were addressed	Page 7/Line 133-137	Methods/Paragraph 5
		(d) ***Cohort study***—If applicable, explain how loss to follow-up was addressed<br>***Case-control study***—If applicable, explain how matching of cases and controls was addressed<br>***Cross-sectional study***—If applicable, describe analytical methods taking account of sampling strategy	NA	NA
		(e) Describe any sensitivity analyses	NA	NA
**Results**				
Participants	13*	(a) Report numbers of individuals at each stage of study—eg numbers potentially eligible, examined for eligibility, confirmed eligible, included in the study, completing follow-up, and analysed	Page 8-9/Line 158-159	Results/Paragraph 1
		(b) Give reasons for non-participation at each stage	Figure 1	Figure 1
		(c) Consider use of a flow diagram	Figure 1	Figure 1
Descriptive data	14*	(a) Give characteristics of study participants (eg demographic, clinical, social) and information on exposures and potential confounders	Page 8-9/Line 161-174	Result/Paragraph 1
		(b) Indicate number of participants with missing data for each variable of interest	Table S2	Table S2
		(c) ***Cohort study***—Summarise follow-up time (eg, average and total amount)	NA	NA
Outcome data	15*	***Cohort study***—Report numbers of outcome events or summary measures over time	Page 9-11/Line175-223	Result/Paragraph 2-5
		Case-control study—Report numbers in each exposure category, or summary measures of exposure	NA	NA
		Cross-sectional study—Report numbers of outcome events or summary measures	NA	NA
Main results	16	(a) Give unadjusted estimates and, if applicable, confounder-adjusted estimates and their precision (eg, 95% confidence interval). Make clear which confounders were adjusted for and why they were included	Page 9-11/Line 175-223	Result/Paragraph 2-5
		(b) Report category boundaries when continuous variables were categorized	NA	NA
		(c) If relevant, consider translating estimates of relative risk into absolute risk for a meaningful time period	NA	NA
Other analyses	17	Report other analyses done—eg analyses of subgroups and interactions, and sensitivity analyses	Page 11/Line 219-223	Result/Paragraph 5
**Discussion**				
Key results	18	Summarise key results with reference to study objectives	Page 11 /Line 225-234	Discussion/ Paragraph 1
Limitations	19	Discuss limitations of the study, taking into account sources of potential bias or imprecision. Discuss both direction and magnitude of any potential bias	Page 15-16/ Line 320-334	Discussion/Paragraph 6

PDF-XChange Editor Click to BUY NOW! www.tracker-software.com

PDF-XChange Editor www.tracker-software.com Click to BUY NOW!

Interpretation	20	Give a cautious overall interpretation of results considering objectives, limitations, multiplicity of analyses, results from similar studies, and other relevant evidence	Page 11-14/ Line 225-319	Discussion/Paragraph 1-5
Generalisability	21	Discuss the generalisability (external validity) of the study results	Page 15-16/ Line 320-334	Discussion/Paragraph 6
**Other information**				
Funding	22	Give the source of funding and the role of the funders for the present study and, if applicable, for the original study on which the present article is based	Page 16-17/ Line 341-346	Acknowledgements

*Give information separately for cases and controls in case-control studies and, if applicable, for exposed and unexposed groups in cohort and cross-sectional studies.

Note: An Explanation and Elaboration article discusses each checklist item and gives methodological background and published examples of transparent reporting. The STROBE checklist is best used in conjunction with this article (freely available on the Web sites of PLoS Medicine at http://www.plosmedicine.org/, Annals of Internal Medicine at http://www.annals.org/, and Epidemiology at http://www.epidem.com/). Information on the STROBE Initiative is available at www.strobe-statement.org.
